# Diphtheria Antitoxin Production and Procurement Practices and Challenges

**DOI:** 10.3201/eid3112.250796

**Published:** 2025-12

**Authors:** Caroline Marshall, William Perea Caro, Alejandro Costa, Lee Lee Ho, Peter J. Gardner, Christophe Guitton, Julien Potet, Erin Sparrow

**Affiliations:** London School of Hygiene & Tropical Medicine Faculty of Epidemiology and Public Health, London, UK (C. Marshall); World Health Organization, Geneva, Switzerland (C. Marshall, W.P. Caro, A. Costa, L.L. Ho, C. Guitton, E. Sparrow); Wellcome Trust, London, UK (P.J. Gardner); Médecins Sans Frontières, Paris, France (J. Potet)

**Keywords:** diphtheria, antitoxin, outbreak management, outbreak response, respiratory illness, bacteria, infectious disease

## Abstract

Treatment of respiratory diphtheria requires prompt administration of equine diphtheria antitoxin (DAT) to neutralize circulating toxin. We conducted surveys of key procurement agencies and manufacturers currently engaged in DAT manufacturing or procurement, along with key informant interviews with developers of monoclonal antibodies. Our findings indicate that prices and availability of DAT vary and that prediction of demand is challenging for both manufacturers and procurement agencies. Substantial concerns were raised over the inability to obtain enough DAT to respond to increasing global outbreaks. Monoclonal antibody developers noted financial challenges in advancing their clinical and manufacturing progress.

Before diphtheria toxoid–containing vaccine was broadly available, diphtheria was a leading cause of childhood deaths. After the introduction of the vaccine, the incidence of diphtheria declined. However, because of factors such as diminished routine vaccination coverage in some settings, outbreaks of diphtheria continue to occur; case-fatality rate (CFR) is ≈30% in nonvaccinated persons, with higher risk for death in young children <5 years of age (*1*,*2*). Cases have risen markedly since the early 2000s. In 2023, the World Health Organization (WHO) received reports of 24,782 cases from all regions, mostly in the African, Eastern Mediterranean, and South-East Asia regions; that total represented a dramatic increase from 10,027 reported cases in 2022 (*3*,*4*). Diphtheria is believed to be underreported in many regions (*5*,*6*).

Current treatment of respiratory diphtheria requires hospitalization, the administration of diphtheria antitoxin (DAT, sometimes referred to as eDAT, which contains polyclonal equine immunoglobulins), and antimicrobial drugs (typically penicillin, erythromycin, or another available macrolide) for a course of 14 days (*7*). The timely administration of DAT can prevent potentially irreversible toxin-related damage, reducing mortality rates by up to 76% (*8*). DAT is included in the WHO model Essential Medicines List; despite its importance, global availability of the product is perilously unreliable. Supply is limited; many producers have ceased production because of unpredictable demand, limited return on investment for continuing production, and the high regulatory requirements necessary to assure safety of blood-derived products. Most countries do not have DAT stockpiles and rely on donations or procurement by United Nations (UN) agencies. Response efforts tend to be agency specific, with no coordinating body or formal communication channels specific to the product. DAT availability is critical for case management and mitigation of diphtheria outbreaks; issues with DAT availability create challenges in preventing further spread of diphtheria (*9*,*10*).

The objectives of this study were to assess the current context and challenges for production and procurement of DAT from the perspectives of manufacturers and procurement agencies and to understand current development of candidate monoclonal antibodies for diphtheria and any challenges impeding their development toward licensure and broader production.

## Methodology

We conducted 3 key activities to collect the necessary data for this assessment. Activity 1 was a literature review and stakeholder mapping exercise of key procurement agencies and manufacturers involved in diphtheria antitoxin procurement and implementation activities for diphtheria outbreaks, by country or region. Activity 2 was surveys of the identified key procurement agencies and manufacturers involved in diphtheria antitoxin supply and procurement and implementation activities for diphtheria outbreaks. Activity 3 was key informant interviews with the developers of monoclonal antibodies currently in development for diphtheria.

### Activity 1—Literature Review and Stakeholder Mapping

To understand the context and recent relevant history of diphtheria antitoxin procurement and inform the selection of the target populations for semistructured interviews, we conducted a landscape analysis of potential WHO stakeholders based on existing engagement trackers and lists and internal desk research. The list included identified persons and organizations working on diphtheria antitoxin manufacturing and procurement.

In May 2023, we conducted a literature review using the search string “diphtheria AND antitoxin AND monoclonal antibody AND treatment” to identify any current monoclonal antibody candidates targeting diphtheria toxin. We supplemented the literature review with a search of https://clinicaltrials.gov using the terms diphtheria and monoclonal. We conducted the literature review only on English-language materials, which is a potential limitation of the research.

In May 2023, we conducted a web search of procurement agencies including UNICEF, WHO, WHO Pan-American Health Organization revolving fund, and Médecins Sans Frontières (MSF) to document publicly available information on DAT procurement. Finally, we developed a list of DAT producers based on information from procurement agencies, existing WHO resources, and through a web search.

### Activity 2—Stakeholder Surveys

During June–July 2023, we conducted surveys of the identified key procurement agencies and manufacturers involved in diphtheria antitoxin procurement and implementation activities for diphtheria outbreaks. The purpose of those surveys was to gain an understanding of current supply and production contexts, forecasting, and general supply chain challenges and opportunities.

### Activity 3—Key Informant Interviews

During August–September 2023, we conducted key informant interviews with MassBiologics (https://www.umassmed.edu/massbiologics) and People for the Ethical Treatment of Animals (PETA) Science Consortium International (https://www.thepsci.eu). The interviews were designed to determine timelines for availability of their monoclonal antibodies (mAbs)–based products, production capacity, and related costs (Appendix).

## Results

### Procurement Agency Survey

Four procurement agencies provided responses to the survey. We had chosen procurement agencies on the basis of known ongoing activities related to diphtheria outbreak response or global procurement responsibilities for outbreak response. Although the United States and some countries in Europe also procure DAT, those supplies are generally limited to small quantities and used only domestically. We do not describe those national-level procurement experiences here, although we note the important role of ministries of health in contributing to health security. In addition, we did not consider private-sector procurement. We obtained further information from internal analysis of procurement data for the World Health Organization (Appendix Table 1). 

In our survey results, procurement agencies identified limited suppliers. We noted significant variability in prices per vial (US $25–$81.89 including administrative fees). Forecasting activities were limited for most procurement agencies; the variability of outbreaks that agencies respond to made demand unpredictable.

As part of the survey, procurement agencies also identified any forecasting activities undertaken internally, and reflections on challenges with current procurement mechanisms for equine DAT. Procurement agencies identified issues within the themes of pricing, quality assurance, product specifications, and manufacturing; pricing variability, lack of guidance such as WHO prequalification or Stringent Regulatory Authority approval, product shelf life, supply and stockpile management, limited suppliers, lack of availability for emergency contexts due to lead time required for manufacturing.

Procurement agencies identified the following areas for further discussion or coordinated support: coordination/implementation of global stockpile; exploration of new products, including support for advancement of products currently in development (e.g., mAbs); exploration and prequalification of new suppliers; forecasting outbreaks and product needs; and advocacy at the country level on the importance of DAT stockpiles and safety stocks.

### Manufacturer Survey

We identified manufacturers from a WHO internal 2017 procurement review indicating current or previous manufacturing activities related to DAT, and from information provided by procurement agencies (*11*). We sent survey invitations to 10 manufacturers; of those, 3 manufacturers provided responses to the survey (Appendix Table 2). Results as presented are based on responses to the survey as well as a supplemental analysis of internal procurement data for WHO that included additional manufacturers Biological E and Premium Serums (Appendix Tables 1, 2).

Seventy percent of manufacturers that had been previously identified did not respond to survey requests, despite numerous reminders. As such, data are limited to the 3 suppliers who responded to the survey, as well as the 3 other manufacturers for which WHO had production capacity and cost data. Those include all manufacturers from where procurement agencies are currently purchasing DAT. As evidenced by survey results, manufacturers confirmed wide variability in prices per vial ($24–$507.20), although manufacturers producing larger volumes tended to report lower prices. Prices were different, in some cases, from those reported by procurement agencies. All 3 manufacturers reported forecasting activities to varying degrees of detail. Manufacturers also reported being challenged by the variability of outbreaks and subsequent demand calculations. As part of the survey, manufacturers also reflected on challenges with current manufacturing and procurement mechanisms for equine DAT. Manufacturers identified challenges within the following themes: availability of starting material (hyperimmune plasma) within the short periods of time needed for product generation for outbreak response; implementation of all current Good Manufacturing Practices (cGMP) requirements; competing priorities (e.g., one manufacturer reported the same facility being used for 11 other equine-derived immunoglobulins); and unpredictable increases in demand. The issues that manufacturers identified for further discussion or coordinated support were updates on global diphtheria cases and better coordination on advance manufacturing.

### Key informant interviews: Monoclonal Antibodies

We identified 2 candidates from the literature review and Clinicaltrials.gov search. The candidates were MassBiologics and PETA Science Consortium International.

#### MassBiologics

We conducted 2 key informant interviews with a representative from MassBiologics on August 23, 2023, and September 6, 2023. In addition to the interview, the company shared overview materials for review for relevant contextual information. Development of S315, a human monoclonal antibody, began at MassBiologics in 2010 (*12*,*13*). S315 is produced through the isolation of antibody-secreting cells in human volunteers boosted with TdVax (*14*), a combination tetanus/diphtheria vaccine. Antibody-producing genes are amplified, synthesized, and screened through antibody binding and toxin neutralization (Figure).

**Figure:**
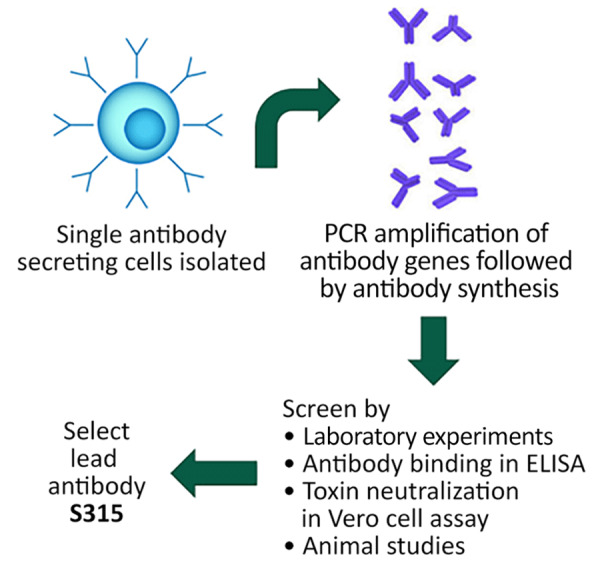
Development of S315, a human monoclonal antibody produced through the isolation of antibody-secreting cells in human volunteers boosted with a combination tetanus/diphtheria vaccine. Antibody-producing genes are amplified, synthesized, and screened through antibody binding and toxin neutralization.

##### S315 Clinical Study Progress

MassBiologics conducted dose-selection study of S315 in a guinea pig model of diphtheria intoxication, in accordance with National Institutes of Health (NIH) minimum requirements potency assays (*15*). Findings from the study suggested a potency estimate of 17 (95% CI 16–21) µg S315/IU DAT for a survival endpoint (*15*). An alternate potency endpoint of 48 (95% CI 38–59) µg/IU was identified after transient limb weakness in some surviving animals was presumed to be a sign of systemic toxicity (*15*).

In 2019, MassBiologics filed an investigational new drug proposal. It has subsequently completed an in-human, randomized, double-blind, dose escalation phase 1 trial of S315 intended to demonstrate safety, tolerability, and pharmacokinetics. The phase 1 study was conducted in a population of healthy adults 18–55 years of age in which S315 was demonstrated to be generally safe and well tolerated. The study found that S315 serum neutralizing activity was an order of magnitude greater than that attained by eDAT (*16*).

##### Challenges in Clinical Study Progress

S315’s progress has been hampered in recent years for several reasons. The emergence and focus on COVID-19 vaccines, therapeutics, and diagnostics, as well as various limitations on in-person activities, caused delays in approvals processes. As of September 2025, the Centers for Disease Control and Prevention (CDC) has approved a protocol for expanded access/compassionate use of S315, but an available manufacturer for GMP treatment courses has not been identified.

Regulatory approval typically requires data collection through a randomized controlled trial or use of Animal Rule Good Laboratory Practices studies. MassBiologics received a Food and Drug Administration decision in November 2023 that acknowledged the challenges of conducting human trials but stated that a guinea pig rescue model is not sufficient; exploration of clinical trials would require a change in process toward a biologic licensing application. The company could not share specific details. 

MassBiologics did investigate opportunities for noninferiority clinical field trials with the WHO and MSF from 2020 forward, comparing monoclonal DAT (mDAT) to equine DAT, but those were limited by the COVID-19 pandemic. Further discussions were held in January 2023 to determine if outbreaks in Niger and Nigeria would warrant use and evaluation of S315, but no decision was made on a clinical field trial (*16*). MassBiologics has identified concerns with holding clinical field trials that include the necessary population size for an adequately powered study (estimated at 674 total, and 337 per treatment group). Adequate population size is influenced by current epidemiology of diphtheria, specifically the variability in scale, location, and duration of outbreaks, which makes planning the logistical elements of a trial and ensuring adequate supply of both mDAT and DAT difficult (*1*,*5*,*17*).

MassBiologics has reviewed WHO 2023 data and identified that, during 2017–2021, Ethiopia, India, Indonesia, Nigeria, Pakistan, Venezuela, and Yemen each reported a median of >150 cases/year (range 164–5,293 cases/year) and as such would potentially fit criteria for conducting a clinical trial (e.g., reported surveillance cases, outbreaks occur regularly, existing infrastructure for clinical trials). However, logistical and study scale elements remain a concern.

##### Challenges in Finance

The development of S315 has been funded by a combination of grants and internal budget at MassBiologics. However, as a state-owned, not-for-profit company, MassBiologics is limited in its resources and its access to financial markets, which has created additional challenges to S315’s timely progress. Although MassBiologics has made several attempts to identify a private-sector partner for co-development (including licensing and manufacturing), no successful partnership has been identified. MassBiologics has also discussed grant support with several philanthropic organizations, multilateral organizations, and nongovernmental organizations about various topics, but those conversations have primarily focused on developing clinical studies and stockpiling S315 and have not resulted in financial support.

#### PETA Science Consortium International

One key informant interview was held on October 4, 2023, with representatives associated with a research project funded by the PETA Science Consortium International. In addition to the interview, the Consortium shared overview materials that we reviewed for relevant contextual information.

PETA Science Consortium International is developing a candidate product for the treatment of diphtheria that is a combination of 2 human recombinant mAbs (*18*). The candidate was developed after the generation of 400 human recombinant antibodies against diphtheria toxin from 2 phage display panning strategies using a human immune library. Narrowing down the identified 400 through various panning and neutralization screening techniques, the researchers further characterized 61 unique antibodies, 35 produced as fully human IgG1 (*18*). The researchers also determined that a 2-mAb cocktail resulted in better neutralizing capacity and higher potency (*18*). With those results, they have established proof of concept and are in the preclinical phase but do not yet have GMP material. They anticipate that the candidate product will be available to move to human trials in future years, pending a development partner and further financing. The consortium is a nonprofit organization and does not expect to have any financial stake in further development of the candidate; in the absence of anticipated profit generation, candidate product development requires further financial support.

##### Challenges in Clinical Progression and Finance

Similar to MassBiologics, as a not-for-profit organization, the consortium has limited resources for comprehensive human trials. The consortium will require financial support from partners. 

## Discussion

The surveys and key informant interviews highlight that prices and availability of DAT vary widely and that prediction of demand is challenging for both manufacturers and procurement agencies. Procurement agencies raised concerns over the inability to obtain sufficient amounts of DAT to respond to increasing global outbreaks (*19*–*26*). Related factors raised included broad variability in pricing, a lack of procurement agency resources available to engage with manufacturers, and the absence of prequalified or stringent regulatory authority–approved DAT. There was no clear indication of why prices varied so widely, although there appeared to be some correlation between larger contract volumes and lower prices, as would be expected in contracting practices.

With respect to product specifications, procurement agencies noted that the product’s relatively short shelf-life resulted in a purchase risk for the organization, and given the lower quantities typically procured in comparison to other public health products and the challenges with supply availability, many procurement agencies were not well placed to take that risk. Suggested ways to mitigate those risks included the coordination of a global stockpile, as was done for cholera vaccines (*27*), from which multiple agencies could draw, which would coordinate demand, introduce supportive financing mechanisms, and increase consistency across prices. Such a stockpile could also mitigate another concern raised, the necessary lead time for manufacturing, which proves challenging for outbreak response purposes. There is also a need to discuss the roles and responsibility for management of such a stockpile, similar to Gavi’s International Coordination Group (ICG) on Vaccine Provision, as well as distribution responsibilities, because diphtheria infections are not a high priority for response compared with other pathogens requiring vaccines or therapeutics. The stockpile approach would also require the definition of transportation requirements and responsibilities as well as funding authorities.

Although manufacturers overall expressed fewer concerns, the lack of response from several manufacturing organizations could mean that there are unexpressed concerns from other manufacturers. Some of the contacted manufacturers could no longer be involved in DAT supply, which would represent a further reduction in DAT availability from several years ago (Appendix). Manufacturers who responded to the survey indicated some forecasting or future estimates for manufacturing but did not identify any substantive ability to expand manufacturing capacity in a timely manner (e.g., surge capacity during outbreaks requiring increased production). Manufacturers also identified GMP requirements as a rate-limiting step for timely surge DAT production that would require increased speed of production. Those requirements should be considered nonnegotiable for procurement; however, there is a potential role for increasing prequalification of suppliers or enabling coordination with suppliers to promote capacity to meet GMP requirements in advance of increases in production needs or surge requirements.

Those solutions might help to mitigate some manufacturing issues, but they do not address other concerns with DAT, namely batch-to-batch variability of product, risk for hypersensitivity or allergic reactions, and timely ability to manufacture in response to outbreaks. Both organizations currently developing a monoclonal antibody product for DAT are making progress in development; however, both are hampered by a lack of available funding for research, lack of clarity around the potential implementation of a randomized control trial in outbreak situations to enable comparison against DAT, and lack of a clear path for manufacturing upon licensure. With respect to a potential clinical trial for mAbs, there is also an ethical concern of carrying out trials for market authorization in geographic locations for which the product may be too expensive, as was noted during clinical trials for Ebola viral disease treatment with mAbs. Although both products show great scientific promise, cost per treatment would likely be substantially higher than that of DAT.

## Conclusions

The introduction of diphtheria vaccine has greatly reduced global incidence of diphtheria and contributed to improved childhood health. However, because of inconsistency in vaccine access, supply, and confidence, childhood immunization rates have waned globally in recent years and diphtheria outbreaks have risen, driving increased but unpredictable DAT demand and causing subsequent issues with consistent and rapid access to sufficient DAT. The importance of maintaining DAT manufacturing, supply, and access for health security of a vaccine-preventable disease should be recognized in the short term in tandem with a long-term understanding of the benefits of DAT replacement by more effective and safe toxin-targeting monoclonal antibodies that offer the promise of a 21st Century solution to DAT limitations.

Transition from DAT to monoclonal antibodies could be accomplished by supplementation of DAT supplies with laboratory-produced mDAT; 2 candidates are currently in clinical development. However, both mDAT candidates face challenges in advancing their clinical and manufacturing progress, such as an unclear value proposition and business case, lack of preferred product characteristics guidelines, lack of clear regulatory guidance, and the challenge of clearly communicating the unmet need in the face of an effective vaccine.

The perspectives we learned through surveys and interviews were invaluable. Our review was limited by nonresponse of some manufacturers, lack of ability to use publications or information in other languages, and lack of historical data about manufacturers no longer producing therapeutics.

Although WHO is maintaining a small stock of DAT to respond to urgent requests, no specific financial and allocation mechanism similar to an ICG has been established for diphtheria antitoxin. We noted a need to reconvene a group of experts drawn from manufacturers, procurement agencies, multilateral agencies, regulatory bodies, and philanthropic organizations. The mission of such a group would be to assess proposed options for viability and develop a shared commitment to increase financing for advancing mDAT product development while ensuring access to sufficient DAT supply.

Appendix Additional information about diphtheria antitoxin production and procurement practices and challenges.
