# Supplementary material for: Diphtheria Antitoxin Production and Procurement Practices and Challenges
Source: Emerg Infect Dis. 2025 Dec;31(12):e250796. doi: 10.3201/eid3112.250796 (PMC12782264; doi:10.3201/eid3112.250796)
Supplement: Appendix — Additional information about diphtheria antitoxin production and procurement practices and challenges. [file 25-0796-Techapp-s1.pdf]

Article DOI: https://doi.org/10.3201/eid3112.250796

EID cannot ensure accessibility for supplementary materials supplied by authors. Readers who have difficulty accessing supplementary content should contact the authors for assistance.

Diphtheria Antitoxin Production and Procurement Practices and Challenges

Appendix

Key Informant Interview Guiding Questions

History through to Market Authorization

- What is your historical development of mAbs for diphtheria? (E.g. years in development, trials)
- What development do you still have to undertake to gain market authorization?
- When is market authorization by U.S. FDA anticipated?

Production capacity/ manufacturing assessments

- Have you undertaken any manufacturing assessments with respect to production capacity and productions costs? If yes:
    - o What is your product unit of measure?
    - o What is your projected ability to produce per year?
    - o What is your current Cost of Goods (CoGs) both by per gram of antibody and per treatment course?
    - o Do you have plans to expand capacity? Or have you identified a manufacturing partner to produce your diphtheria mAb in the future?
    - o What is the current production platform used (CHO cell, stainless steel?)
    - o Have you considered other production technologies such as continuous processing, innovative bioreactors (e.g. UNIVERCELLS)?

Market Costs and Procurement Agency Relationships

- Have you established specifics with respect to costs/ minimum orders/ etc.?
- Have you undertaken any demand assessments with respect to how much you expect to need to produce?

• Have you had discussions already with procurement agencies and/ or countries about supply?

If yes, are you willing to share any contextual details about this?

General Questions and Challenges

• Have you experienced any production challenges thus far?

• Do you anticipate new challenges arising? If so, which ones?

• How does the current global health landscape support or challenge your ability to produce and distribute this product?

• Are there any specific changes you would make to this landscape? E.g. financing mechanisms

• Is there anything else you'd like to share?

Procurement Agency Survey Questions

1. What is the number of vials of DAT procured over the last 5 years, by year?

2. Which suppliers do you procure from?

3. What price does each supplier charge you? Please indicate if this has changed over time.

4. What volume have you procured from each supplier, per year, for each of the past 5 years?

5. Which countries have you procured DAT on behalf of?

6. Have you undertaken forecasting activities for your DAT supply needs for the near future?

    o If so, how far have you forecasted?

7. Has your forecast highlighted any potential or anticipated supply challenges?

8. Which countries have you engaged in forecasting activities for, and what estimates have you calculated? If willing, please describe results below.

9. Would you be willing to share further information on your forecasting models?

10. What challenges have you encountered generally in your procurement processes?

11. Do you have any other feedback you would like to share with us?

Manufacturer Survey Questions

1. Which agencies and/ or countries do you supply DAT to?

2. What price do you charge per vial? Please indicate if this has changed over time.

3. What volume have you provided for each agency/ country, per year, for each of the past 5 years, if permitted to share?

4. Have you undertaken forecasting activities for your DAT production requirements for the near future?

   a. If so, how far have you forecasted?

5. Has your forecast highlighted any potential or anticipated production challenges?

6. What challenges have you encountered generally in your production processes?

Historical Procurement Data for Known Manufacturers (2017)

Appendix Table 1. Survey and data collection results for procurement agencies, 2018–2022

Agency	Doses procured in last 5 years	Countries of use	Supplier	Forecasting activities
Médecins Sans Frontières (MSF)	Estimated:<br>2022: 2,500 doses<br>2021: 800 doses<br>2020: 1,300 doses<br>2019: 3,300 doses<br>2018: 4,000 doses<br>Total: 11,900	Bangladesh, Kenya, Niger, Nigeria, Pakistan, Somalia, Venezuela, Yemen	Premium Serums	Undertaken for MSF's countries of focus, Taking into consideration past consumption and possible emerging epidemics
United Nations Children's Fund (UNICEF)	4,700 total	Myanmar, Pakistan	Premium Serums	Limited; forecasting is mainly done for products that are considered strategic to UNICEF
Pan American Health Organization (PAHO)	2022: 1,650<br>2021: 1,029<br>2020: 1,323<br>2019: 1,325<br>2018: 1,460<br>Total: 6,787	Bolivia, Brazil, Colombia, Curacao, Dominican Republic, Ecuador, Haiti, Honduras, Panama, Perú, Uruguay, Venezuela	2022: Instituto Butantan<br>2018–2021: VINS Bioproducts LTD	One year (January–December 2024), only 3 of 41 AMRO countries participated in forecasting.
World Health Organization (WHO)	2023: 26,057<br>2022: 8,470<br>2020: 5,875<br>2019: 3,500<br>2018: 7,100<br>Total: 51,002	Bangladesh, Djibouti, Indonesia, Myanmar, Nigeria, Pakistan, Switzerland, UAE, Yemen	Premium Serums and Haffkine<br>Also obtain supplies from Biological E.	Ongoing based on outbreaks

Appendix Table 2. Survey and data collection results for procurement agencies*

Manufacturer	5-year production, no.	Countries/ agencies supplied to	Volumes produced per country/ agency	Forecasting
BulBio	2022: 1,453 ampoules	Bulgaria, Latvia	2023: 38 amps Bulgaria, 400 amps Latvia	Yes – next 3 years
Butantan	2023:3,066 vials (Jan-Jun)<br>2022: 6,423 vials<br>2021: null (2020 production supplied 2021 demand)<br>2020: 1,925 vials<br>2019: 4,044 vials	MoH Brazil;<br>PAHO – Bermuda, Bolivia, Colombia, Cuba, Dominican Republic, Honduras, Panama, Uruguay;<br>UNICEF – Kazakhstan, Myanmar;<br>Other countries – Austria, Canada, Chile, Belgium, England, France, Germany, Netherlands, Switzerland, USA;<br>MSF	Estimates from 2021–2023:<br>MoH Brazil, 1,000 vials<br>PAHO, 1,500 vials<br>UNICEF, 1,500 vials<br>Other countries, around 500 vials<br>MSF, 500 vials	Yes – estimated 5,000 vials per year. Capacity is to produce one batch of 1,200–1,300 vials every 3/4 months.
Vins Bioproducts Limited	2022–23: 27,551 vials<br>2021–22: 25,274 vials<br>2020–21: 31,286 vials<br>2019–20: 25,771 vials<br>2018–19: 24,296 vials<br>Total: 134,178	Algeria, Canada, Haiti, Indonesia, Jordan, Saudi Arabia, Thailand, Tunisia, Ukraine, several state governments in India	NA	7,000–8,000/ month production capacity
Premium Serums	24,000–30,000 per year (2,000–2,500 vials/month)†	WHO	NA	2,000 / batch, can run 2 batches together / month, 60 days lead time required
Biological E	36,000 per year (3,000/month)†	WHO	NA	Lead time 6 months for first shipment, 11 weeks for subsequent
Haffkine Bio Pharmaceutical Corporation	20,000 per year†	NA	NA	Lead time 3 months

*Each vial contains 10,000 IU of DAT immunoglobulin antigen-binding fragments. Dosage recommendations vary depending on clinical presentation and are the same for pediatric and adult populations: pharyngeal or laryngeal disease of 2 days duration, 20,000–40,000 IU recommended (2–4 vials); nasopharyngeal disease, 40,000–60,000 IU recommended (4–6 vials); extensive disease of 3 or more days duration, 80,000–100,000 IU recommended (8–10 vials); skin lesions only (rare), 20,000–40,000 IU recommended (2–4 vials)(11). NA, not available.
†Indicates estimated capacity.
